# Role of Arbuscular Mycorrhizal Fungi in Plant Growth Regulation: Implications in Abiotic Stress Tolerance

**DOI:** 10.3389/fpls.2019.01068

**Published:** 2019-09-19

**Authors:** Naheeda Begum, Cheng Qin, Muhammad Abass Ahanger, Sajjad Raza, Muhammad Ishfaq Khan, Muhammad Ashraf, Nadeem Ahmed, Lixin Zhang

**Affiliations:** ^1^College of Life Sciences, Northwest A&F University, Yangling, China; ^2^College of Natural Resources and Environment, Northwest A&F University, Yangling, China; ^3^Department of Weed Science, The University of Agriculture, Peshawar, Pakistan; ^4^University of Agriculture Faisalabad, Pakistan; ^5^Department of Botany, Mohi-Ud-Din Islamic University Azad Jammu and Kashmir, Pakistan

**Keywords:** arbuscular mycorrhizal fungi, plant growth, abiotic factors, stress tolerance, mineral nutrition

## Abstract

Abiotic stresses hamper plant growth and productivity. Climate change and agricultural malpractices like excessive use of fertilizers and pesticides have aggravated the effects of abiotic stresses on crop productivity and degraded the ecosystem. There is an urgent need for environment-friendly management techniques such as the use of arbuscular mycorrhizal fungi (AMF) for enhancing crop productivity. AMF are commonly known as bio-fertilizers. Moreover, it is widely believed that the inoculation of AMF provides tolerance to host plants against various stressful situations like heat, salinity, drought, metals, and extreme temperatures. AMF may both assist host plants in the up-regulation of tolerance mechanisms and prevent the down-regulation of key metabolic pathways. AMF, being natural root symbionts, provide essential plant inorganic nutrients to host plants, thereby improving growth and yield under unstressed and stressed regimes. The role of AMF as a bio-fertilizer can potentially strengthen plants’ adaptability to changing environment. Thus, further research focusing on the AMF-mediated promotion of crop quality and productivity is needed. The present review provides a comprehensive up-to-date knowledge on AMF and their influence on host plants at various growth stages, their advantages and applications, and consequently the importance of the relationships of different plant nutrients with AMF.

## Introduction

Arbuscular mycorrhizal fungi (AMF) facilitate host plants to grow vigorously under stressful conditions by mediating a series of complex communication events between the plant and the fungus leading to enhanced photosynthetic rate and other gas exchange-related traits (Birhane et al., 2012), as well as increased water uptake. Numerous reports describe improved resistance to a variety of stresses including drought, salinity, herbivory, temperature, metals, and diseases due to fungal symbiosis (Rodriguez et al., 2008; Ahanger et al., 2014; Salam et al., 2017). Nearly 90% of plant species including flowering plants, bryophytes, and ferns can develop interdependent connections with AMF (Zhu et al., 2010a; Ahanger et al., 2014). AMF form vesicles, arbuscules, and hyphae in roots, and also spores and hyphae in the rhizosphere. Formation of hyphal network by the AMF with plant roots significantly enhances the access of roots to a large soil surface area, causing improvement in plant growth (Bowles et al., 2016). AMF improve plant nutrition by increasing the availability as well as translocation of various nutrients (Rouphael et al., 2015). AMF improve the quality of soil by influencing its structure and texture, and hence plant health (Zou et al., 2016; Thirkell et al., 2017). Fungal hyphae can expedite the decomposition process of soil organic matter (Paterson et al., 2016). Furthermore, mycorrhizal fungi may affect atmospheric CO_2_ fixation by host plants, by increasing “sink effect” and movement of photo-assimilates from the aerial parts to the roots. Keeping in view the importance of AMF and the research advancements related to their applications in agriculture, the present review focuses on the role of AMF as bio-fertilizers in the regulation of plant growth and development with improved nutrient uptake under stressful environments, and the level to which AMF can enhance plant growth under stressful environments.

## Background of Arbuscular Mycorrhizal Fungi

AMF are soil-borne fungi that can significantly improve plant nutrient uptake and resistance to several abiotic stress factors (Sun et al., 2018). A majority of the species of AMF belong to the sub-phylum Glomeromycotina, of the phylum Mucoromycota (Spatafora et al., 2016). Four orders of AMF, namely, Glomerales, Archaeosporales, Paraglomerales, and Diversisporales, have been identified in this sub-phylum that also include 25 genera (Redecker et al., 2013). They are obligate biotrophs and ingest plant photosynthetic products (Bago et al., 2000) and lipids to accomplish their life cycle (Jiang et al., 2017). AMF-mediated growth promotion is not only by improving water and mineral nutrient uptake from the adjoining soil but also by safeguarding the plants from fungal pathogens (Smith and Read, 2008; Jung et al., 2012). Therefore, AMF are vital endosymbionts playing an effective role in plant productivity and the functioning of the ecosystem. They are of key importance for sustainable crop improvement (Gianinazzi et al., 2010).

## Characteristics of AMF Symbiosis

**Figure 1:**
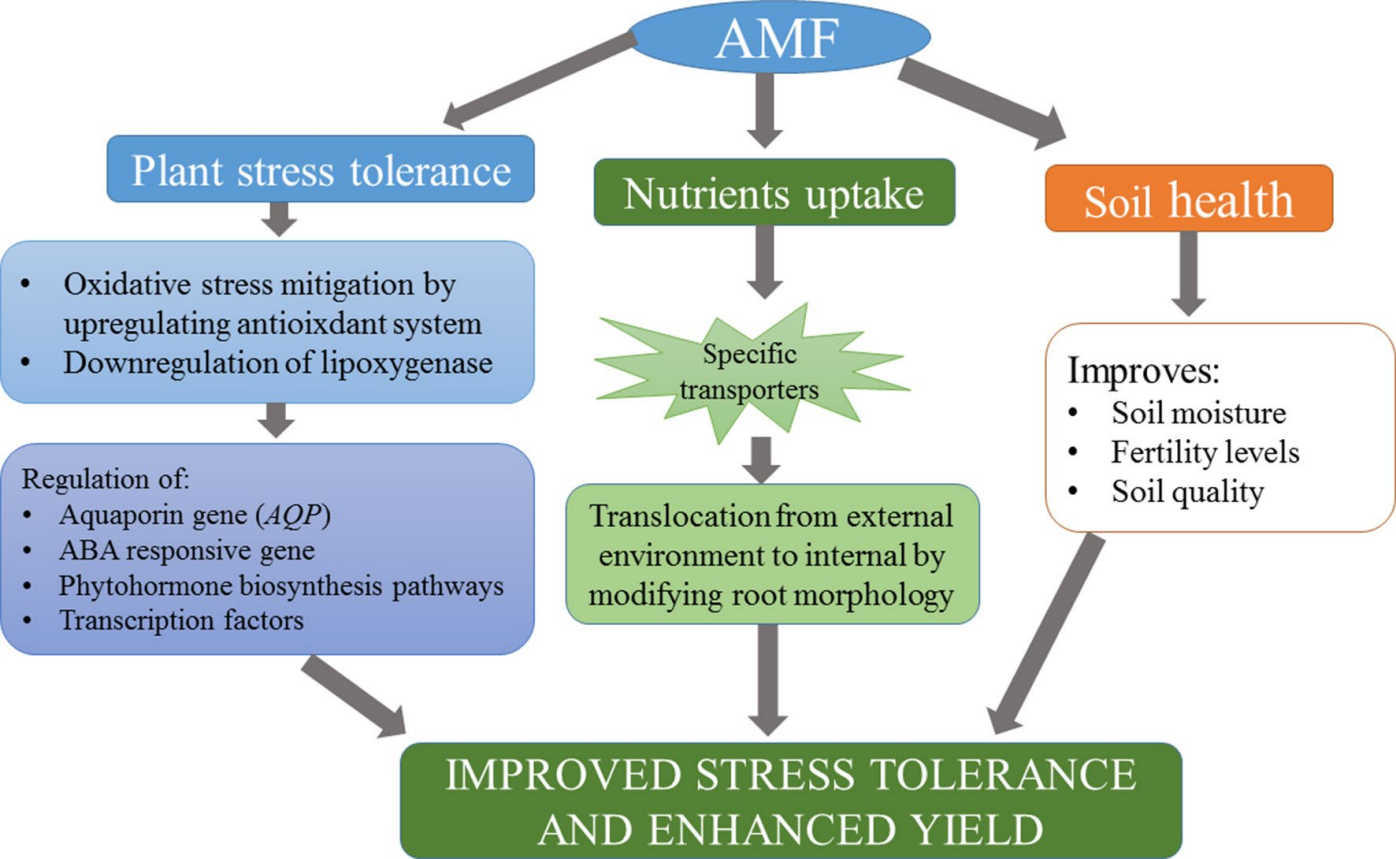
A diagrammatic representation of mycorrhizal functions to regulate various processes in the ecosystem and plant growth promotion under abiotic stress condition.

The symbiosis of AMF with plants had been reported 400 million years ago (Selosse et al., 2015). Such types of links are established as a succession of biological processes, which lead to a variety of useful effects in both natural ecosystem and agricultural biotas (Van der Heijden et al., 2015). The symbiotic association of AMF is a classic example of mutualistic relationship, which can regulate the growth and development of plants. The mycelial network of fungi extends under the roots of the plant and promotes nutrient uptake that is otherwise not available. The fungal mycelium colonizes roots of many plants even if they belong to different species, resulting into a common mycorrhizal network (CMN). This CMN is considered as a primary component of the terrestrial ecosystem with its significant effects on different plant communities, particularly on invasive plants (Pringle et al., 2009) and the fungal-mediated transport of phosphorus (P) and nitrogen (N) to plants (Smith and Read, 2008). Moreover, communal nutrients also relocate from fungi to the plant, along with other related effects, which is probably why AMF improve plant tolerance to biotic and abiotic factors (Plassard and Dell, 2010). They have the ability to improve characteristics of soil and consequently encourage plant development in normal as well as in stressful circumstances (Navarro et al., 2014; Alqarawi et al., 2014a; Alqarawi et al., 2014b). AMF colonization improves tolerance of plants to stressful cues by bringing about several changes in their morpho-physiological traits (Alqarawi et al., 2014a; Alqarawi et al., 2014b; Hashem et al., 2015). AMF are considered as natural growth regulators of a majority of terrestrial flora. AMF are used as bio-inoculants, and researchers encourage their use as prominent bio-fertilizers in sustainable crop productivity (Barrow, 2012). Furthermore, AMF-inoculated soil forms more constant masses and significantly higher extra-radical hyphal mycelium than do the non-AMF-treated soils (Syamsiyah et al., 2018). Glomalin-related soil protein (GRSP) is believed to maintain water content in soils exposed to different abiotic stresses (Wu et al., 2014), which later on regulates water frequencies between soil and plants, automatically triggering plant development. Glomalin contains 30–40% C and its related compounds that safeguard soil from desiccation by enhancing the soil water holding capacity (Sharma et al., 2017). Growth-related functions, for example, stomatal conductance, leaf water potential, relative water content (RWC), PSII efficiency, and CO_2_ assimilation are affected by AMF inoculation (He et al., 2017; Chandrasekaran et al., 2019). AMF also help improve water stress tolerance by physiological alteration of the above-ground organs and tissues (Bárzana et al., 2012). Furthermore, inoculation of AMF improves the accumulation of dry matter and enhances water moisture uptake, consequently improving plant tolerance against stresses like drought and salinity. Exploitation of AMF for plant growth in various biological ecosystems can contribute greatly to organic culturing for growth promotion and yield maximization (**Figure 1**).

## AMF as a Bio-fertilizer

Bio-fertilizers are a mixture of naturally occurring substances that are used to improve soil fertility. These fertilizers are very useful for soil health as well as for plant growth and development (Sadhana, 2014). Different research studies conducted on AMF during the past two decades have highlighted their countless benefits on soil health and crop productivity. Therefore, it is widely believed that AMF could be considered as a replacement of inorganic fertilizers in the near future, because mycorrhizal application can effectively reduce the quantitative use of chemical fertilizer input especially of phosphorus (Ortas, 2012). Continuous use of inorganic fertilizers, herbicides, and fungicides has caused various problems to soil, plants, and human health, through their damaging impact on the quality of food products, soil health, and air and water systems (Yang et al., 2004). It is believed that AMF can possibly lower down the use of chemical fertilizers up to 50% for best agricultural production, but this estimate depends on the type of plant species and the prevalent stressful regimes (**Table 1**).

**Table 1:** Observed responses of plants to the inoculation application of AMF on host species exposed to various abiotic stress treatments.

Stress	Host species	Fungus species	Observed responses	References
Drought	*Glycine max* L.	*AMF*	Enhanced leaf proline, photosynthesis, leaf area index, relative growth rate, fresh weight, and dry weight of seeds	Pavithra and Yapa (2018)
Drought	*Poncirus trifoliata*	*Funneliformis mosseae, Paraglomus occultum*	Increased hyphal length, hyphal water absorption rate, and leaf water potential	Zhang et al. (2018a)
Drought	*Olea europaea*	*AMF*	Alleviated drought impact and increased turgor potential (Ψp) and mineral uptake	Sara et al. (2018)
Drought	*Triticum aestivum* L.	*Glomus mosseae, Glomus fasciculatum, Gigaspora decipiens*	Increased plant growth parameters, and total chlorophyll pigments	Pal and Pandey (2016)
Drought	*Digitaria eriantha*	*Rhizophagus irregularis*	Increased shoot dry matter, stomatal conductance, lipid peroxidation, H_2_O_2_ in shoot and root	Pedranzani et al. (2016)
Drought	*Triticum aestivum*	*Glomus mosseae*	Increased osmotic potential, chlorophyll content and fluorescence, activities of antioxidant enzymes, ascorbic acid, enzymes of N and P metabolism, and contents of N, P, and K	Rani (2016)
Drought	*Triticum durum*	*Rhizophagus intraradices*	Higher grain biomass, and higher contents of copper, iron, manganese, zinc and gliadins in grains	Goicoechea et al. (2016, 2017)
Drought	*Ipomoea batatas*	*Glomus spp.*	Proline and soluble sugars adjust osmotic potential	Yooyongwech et al. (2016)
Drought	*Saccharum arundinaceum* Retz.	*Glomus spp.*	Increased leaf water uptake, and levels of metabolites, phenolics, ascorbic acid, glutathione, antioxidant enzymes, chlorophyll fluorescence, and plant biomass	Mirshad and Puthur (2016)
Drought	*Zea mays*	*Rhizophagus intraradices, strain BGCBJ09*	Increased plant dry weight, uptake of P, N, K, and Mg in shoot, and water use efficiency	Zhao et al. (2015)
Drought	*Lettuce and tomato*	*Rhizophagus irregularis,* *Glomus intraradices*	Increased biomass production, efficiency of photosystem II, ABA accumulation and synthesis, and strigolactone production	Ruiz-Lozano et al. (2015)
Drought	*Pelargonium graveolens*	*Rhizophagus intraradices, Funneliformis mosseae*	Improved nutrient concentration, plant biomass, and essential oil content, and glomalin related soil proteins (GRSP)	Amiri et al. (2015)
Drought	*Fragaria ananassa*	*F. mosseae BEG25, F. geosporus BEG11*	Increased shoot and root fresh weights, WUE, and plant survival	Boyer et al. (2014)
Drought	*Robinia pseudoacacia* L.	*Funneliformis mosseae and Rhizophagus intraradices*	Increased dry biomass, WUE, and net photosynthetic rate	Yang et al. (2014)
Drought	Glycine max	*Septoglomus constrictum, Glomus spp.* including *Glomus aggregatum*	Improved water content and P and N levels	Grümberg et al. (2015)
Drought	*Antirrhinum majus* L.	*Glomus deserticola*	Increased shoot and root diameter, shoot length, leaf area, leaf number per plant, water content, Chl content, and proline	Asrar et al. (2012)
Drought	Vigna subterranea	*Glomus intraradices, Gigaspora gregaria, Scutellospora gregaria*	Increased mineral content, soluble sugars, and acid phosphatase, but reduced proline content	Tsoata et al. (2015)
Drought	Hordeum vulgare	*Glomus intraradices*	Increased root volume, P content, and activity of phosphatase enzyme	Bayani et al. (2015)
Heat	*Triticum aestivum* L.	*Rhizophagus irregularis, Funneliformis mosseae, Funneliformis geosporum, Claroideoglomus claroideum*	Increased grain number, nutrient allocation, and nutrient composition in root	Cabral et al. (2016)
High temperature	*Zea mays*	*Rhizophagus intraradices, Funneliformis mosseae, F. geosporum*	Increased leaf length, plant height, leaf number, chlorophyll a, photosynthetic rate, stomatal conductance, and transpiration rate	Mathur et al. (2016)
High temperature	*Solanum lycopersicum*	*Rhizophagus irregularis*	Enhanced photosynthetic capacity, root hydraulic conductivity or aquaporin abundance and phosphorylation status	Calvo-Polanco et al. (2016)
Metal—General	*Sesbania rostrata*	*Glomus mosseae*	Stimulated formation of root nodules, and increased N and P contents	Lin et al.(2007)
Metals—Cadmium	*Trigonella foenum-graecum* L.	*Glomus monosporum, G. clarum, Gigaspora nigra, and Acaulospora laevis*	Increased antioxidant enzymes activities and malondialdehyde content.	Abdelhameed and Rabab (2019)
Metals—Cadmium and zinc	*Cajanus cajan* L.	*Rhizophagus irregularis*	Improved root biomass, nutrient status (P, N, Mg, Fe.), and proline biosynthesis	Garg and Singh (2017)
Salinity	*Cucumis sativus* L.	*Glomus etunicatum, Glomus intraradices, Glomus mosseae*	Increased biomass, photosynthetic pigment synthesis, and enhanced antioxidant enzymes	Hashem et al. (2018)
Salinity	*Solanum lycopersicum* L.	*Rhizophagus irregularis*	Enhanced shoot FW, leaf area, leaf number, root FW, and levels of growth hormones	Khalloufi et al. (2017)
Salinity	*Oryza sativa* L.	*Claroideoglomus etunicatum*	Improved quantum yield of PSII photochemistry, net photosynthetic rate, stomatal conductance	Porcel et al. (2015)
Salinity	*Aeluropus littoralis*	*Claroideoglomus etunicatum*	Increased shoot and root dry mass, stomatal conductance, soluble sugars, free α-amino acids, and Na^+^ and K^+^ uptake	Hajiboland et al. (2015)
Salinity	*Solanum lycopersicum* L.	*Glomus intraradices*	Improved dry matter, ion uptake, growth parameters, and chlorophyll content	Hajiboland et al. (2010)
Salinity	*Acacia nilotica*	*Glomus fasciculate*	Improved root and shoot biomass as well as P, Zn, and Cu contents	Giri et al. (2007)
Salinity-alkali	*Leymus chinensis*	*Glomus mosseae*	Increased colonization rate, seedling weight, water contents, and both P and N.	Jixiang et al. (2017)

## AMF and Mineral Nutrition

Excessive land use may have a drastic impact on the overall biodiversity, which in turn may affect the ecosystem function as shown by several reports (Smith and Read, 1997; Balliu et al., 2015; Nouri et al., 2015; Wagg et al., 2015). A prominent role of such symbiotic relationship is to transfer nutrients, for example, organic carbon (C), in the form of lipids and sugars (Jiang et al., 2017; Luginbuehl et al., 2017). AMF colonization is widely believed to stimulate nutrient uptake in plants (**Table 1**). It is evident that inoculation of AMF can enhance the concentration of various macro-nutrients and micro-nutrients significantly, which leads to increased photosynthate production and hence increased biomass accumulation (Chen et al., 2017; Mitra et al., 2019). AMF have the capability to boost the uptake of inorganic nutrients in almost all plants, specifically of phosphate (Smith et al., 2003; Nell et al., 2010). AMF are also very effective in helping plants to take up nutrients from the nutrient-deficient soils (Kayama and Yamanaka, 2014). Apart from the macronutrients, AMF association has been reported to increase the phyto-availability of micronutrients like zinc and copper (Smith and Read, 1997). AMF improve the surface absorbing capability of host roots (Bisleski, 1973). Experimental trials on tomato plants inoculated with AMF have shown increased leaf area, and nitrogen, potassium, calcium, and phosphorus contents, reflecting enhanced plant growth (Balliu et al., 2015). AMF develop symbiosis with roots to obtain essential nutrients from the host plant and consequently provide mineral nutrients in return, for example, N, P, K, Ca, Zn, and S. Thus, AMF provide nutritional support to the plants even under inappropriate conditions inside the root cells. AMF produce fungal structures like arbuscules, which assist in exchange of inorganic minerals and the compounds of carbon and phosphorus, ultimately imparting a considerable vigor to host plants (Li et al., 2016b; Prasad et al., 2017). Therefore, they can significantly boost the phosphorus concentration in both root and shoot systems (Al-Hmoud and Al-Momany, 2017). Under phosphorus-limited conditions, mycorrhizal association improves phosphorus supply to the infected roots of host plants (Bucher, 2007). For example, Pi uptake rate was markedly improved in the AMF-colonized maize plants (Garcés-Ruiz, 2017). Increased photosynthetic activities and other leaf functions are directly related to improved growth frequency of AMF inoculation that is directly linked to the uptake of N, P, and carbon, which move towards roots and promote the development of tubers. It has been observed that AMF maintain P and N uptake ultimately helping in plant development at higher and lower P levels under different irrigation regimes (Liu et al., 2014; Liu et al., 2018). For example, mycorrhizal symbiosis positively increased the concentrations of N, P, and Fe in *Pelargonium graveolens* L. under drought stress (Amiri et al., 2017). Gomez-Bellot et al. (2015) reported improved levels of P, Ca, and K in *Euonymus japonica* under salinity stress due to instant fungus attachment. In another study, AMF-inoculated *Pistachio* plants exhibited high levels of P, K, Zn, and Mn under drought stress (Bagheri et al., 2012). In addition, AMF inoculation improved P and N contents in *Chrysanthemum morifolium* plant tissues (Wang et al., 2018) and increased seedling weight by improving water content and intercellular CO_2_, P, and N contents in *Leymus chinensis* (Jixiang et al., 2017).

It is believed that AMF improve the uptake of almost all essential nutrients and contrarily decrease the uptake of Na and Cl, leading to growth stimulation (Evelin et al., 2012). The extra-radical mycelium (ERM) can effectively improve nutrient uptake, thereby improving plant growth and development (Lehmann and Rillig, 2015). Nitrogen (N), being a main source of soil nutrition, is a well-known mineral fertilizer, even in those areas where there are enough livestock and farm-yard manure (FYM). Many scientists have reported the role of AMF in uptake of soil nutrients, especially of N and P, which can effectively promote the growth of host plants (Smith et al., 2011). In higher plants and some crops, N is a premier growth limiting factor. Several studies have explained that AMF have the ability to absorb and transfer N to the nearby plants or host plants (Hodge and Storer, 2015; Battini et al., 2017; Turrini et al., 2018). Zhang et al. (2018a) have demonstrated AMF mediated increased allocation of shoot biomass to panicles and grains through increased N and P redistribution to panicles particularly under low fertilizer levels. Translocation of N into seeds is enhanced from heading to maturity. AMF after establishing symbiosis produce extensive underground extra-radical mycelia ranging from the roots up to the surrounding rhizosphere, thereby helping in improving the uptake of nutrients specifically N (Battini et al., 2017). The interaction of salinity stress and AMF significantly affects the concentrations of P and N and the N:P ratio in plant shoots (Wang et al., 2018). Recently, it has been reported that native AMF treatments produce significant alterations in the N contents of crop plants (Turrini et al., 2018).

It has been widely accepted that fungi have the ability to take substantial amount of N from dead and decomposed material that later increases their fitness to grow and stay alive. Apart from this, large biomass and increased N requirements for AMF render them the main stakeholder of global N pool that is equivalent in scale to fine roots. Thus, they play a pivotal role in the N cycle (Hodge and Fitter, 2010). The AMF extra-radical hyphae can absorb and assimilate inorganic N (Jin et al., 2005). Several studies have shown that approximately 20–75% of the total N uptake of AM plants can be transferred by the AMF to their hosts (Tanaka and Yano, 2005; Govindarajulu et al., 2005; Ahanger et al., 2014; Hameed et al., 2014; Hashem et al., 2018). Increased N in AMF-colonized plants evidently results in higher chlorophyll contents, as chlorophyll molecules can effectively trap N (De Andrade et al., 2015). Other evidences favoring the AMF-mediated improvement in plant N nutrition can also be seen in the literature (Courty et al., 2015; Bucking and Kafle, 2015; Corrêa et al., 2015). AMF inoculation improves C and N accumulation and N assimilation under ambient and elevated CO_2_ concentrations (Zhu et al., 2016). For example, in olive plants, AMF were reported to improve growth, accumulation of micro-nutrients and macro-nutrients, and their allocation in the plantlets grown under increased levels of Mn (Bati et al., 2015).

Enhancement of plant nutrition and maintenance of Ca^2+^ and Na^+^ ratio are the significant dynamic attributes that help improve beneficial aspects of AMF colonization on overall plant performance (Evelin et al., 2012; Abdel Latef and Miransari, 2014). Improved growth and levels of protein, Fe, and Zn were found in mycorrhizal chickpea (Pellegrino and Bedini, 2014). Moreover, different reports have shown enhanced activity of a K^+^ transporter in the mycorrhizal roots of *Lotus japonicus* (Guether et al., 2009; Berruti et al., 2016). Moreover, two meta-analysis reports that appeared a few years ago showed the role of mycorrhizal symbiosis to various micro-nutrients in crops (Lehmann et al., 2014; Lehmann and Rillig, 2015; as reviewed by Berruti et al., 2016). Asrar et al. (2012) reported that the specified fungal association enhanced the contents of macronutrients such as N, P, K, Ca, and Mg of *Antirrhinum majus* under drought. AMF also proved to be effective in restricting the high accumulation of Na, Mn, Mg, and Fe in roots (Bati et al., 2015). Several studies conducted during the last few years have shown that AMF, such as *Glomus mosseae* and *Rhizophagus irregularis* exhibited improved heavy metal translocation in the shoot (Zaefarian et al., 2013; Ali et al., 2015). Micronutrients such as Zn and Cu being diffusion limited in soils are absorbed by plants with the help of mycorrhizal hyphae.

## AMF and Plant Yield

Beneficial rhizosphere microorganisms not only can improve the nutrient status of crops, as described above, but also can enhance the quality of crops. For example, AMF-colonized strawberry exhibited increased levels of secondary metabolites resulting in improved antioxidant property (Castellanos-Morales et al., 2010). AMF can enhance the dietary quality of crops by affecting and production of carotenoids and certain volatile compounds (Hart et al., 2015). Bona et al. (2017) observed beneficial effects of AMF on the quality of tomatoes. In another study, Zeng et al. (2014) have reported increased contents of sugars, organic acids, vitamin C, flavonoids, and minerals due to *Glomus versiforme* resulting in enhanced citrus fruit quality. Mycorrhizal symbiosis induces enhanced accumulation of anthocyanins, chlorophyll, carotenoids, total soluble phenolics, tocopherols, and various mineral nutrients (Baslam et al., 2011). AMF have been employed in a large-scale field production of maize (Sabia et al., 2015), yam (Lu et al., 2015), and potato (Hijri, 2016), confirming that AMF possess a considerable potential for enhancing crop yield. AMF can also enhance the biosynthesis of valuable phytochemicals in edible plants and make them fit for healthy food production chain (Sbrana et al., 2014; Rouphael et al., 2015).

Rouphael et al. (2015) have reported that the abiotic stress mitigation by AMF could occur through maintenance of soil pH, thereby protecting its horticultural value. In addition, AMF can also play a critical role in improving the resistance of plants to stressful environments, as described below.

## AMF and Abiotic Stresses

### Drought

Drought stress affects plant life in many ways; for example, shortage of water to roots reduces rate of transpiration as well as induces oxidative stress (Impa et al., 2012; Hasanuzzaman et al., 2013). Drought stress imparts deleterious effects on plant growth by affecting enzyme activity, ion uptake, and nutrient assimilation (Ahanger and Agarwal, 2017; Ahanger et al., 2017a). However, there is a strong evidence of drought stress alleviation by AMF in different crops such as wheat, barley, maize, soybean, strawberry, and onion (Mena-Violante et al., 2006; Ruiz-Lozano et al., 2015; Yooyongwech et al., 2016; Moradtalab et al., 2019). Plant tolerance to drought could be primarily due to a large volume of soil explored by roots and the extra-radical hyphae of the fungi (Gianinazzi et al., 2010; Orfanoudakis et al., 2010; Gutjahr and Paszkowski, 2013; Zhang et al., 2016).

Such a symbiotic association is believed to regulate a variety of physio-biochemical processes in plants such as increased osmotic adjustment (Kubikova et al., 2001), stomatal regulation by controlling ABA metabolism (Duan et al., 1996), enhanced accumulation of proline (Ruiz-Sánchez et al., 2010; Yooyongwech et al., 2013), or increased glutathione level (Rani, 2016). Symbiotic relationship of various plants with AMF may ultimately improve root size and efficiency, leaf area index, and biomass under the instant conditions of drought (Al-Karaki et al., 2004; Gholamhoseini et al., 2013). Moreover, AMF and their interaction with the host plant are helpful in supporting plants against severe environmental conditions (Ruiz-Lozano, 2003; **Table 1**). The AMF symbiosis also results in enhanced gas exchange, leaf water relations, stomatal conductance, and transpiration rate (Morte et al., 2000; Mena-Violante et al., 2006). AMF can facilitate ABA responses that regulate stomatal conductance and other related physiological processes (Ludwig-Müller, 2010). Recently, Li et al. (2019) have demonstrated AMF-mediated enhancement in growth and photosynthesis in C_3_ (*Leymus chinensis*) and C_4_ (*Hemarthria altissima*) plant species through up-regulation of antioxidant system.

### Salinity

It is widely known that the soil salinization is an increasing environmental problem posing a severe threat to global food security. Salinity stress is known to suppress growth of plants by affecting the vegetative development and net assimilation rate resulting in reduced yield productivity (Hasanuzzaman et al., 2013; Ahanger et al., 2017a). It also promotes the excessive generation of reactive oxygen species (Ahmad et al., 2010; Ahanger and Agarwal, 2017; Ahanger et al., 2017b; Ahanger et al., 2018). Attempts are being made to explore potential means of achieving enhanced crop production under salt affected soils. One such potential means is the judicious use of AMF for mitigating the salinity-induced adverse effects on plants (Santander et al., 2019). Several research studies have reported the efficiency of AMF to impart growth and yield enhancement in plants under salinity stress (Talaat and Shawky, 2014; Abdel Latef and Chaoxing, 2014; **Table 1**). El-Nashar (2017) reported that AMF enhanced growth rate, leaf water potential, and water use efficiency of the *Antirrhinum majus* plants. Recently, Ait-El-Mokhtar et al. (2019) have reported the beneficial effects of AMF symbiosis on physiological parameters such as photosynthetic rate, stomatal conductance, and leaf water relations under saline regimes. AMF significantly alleviated the deleterious effects on photosynthesis under salinity stress (Sheng et al., 2011). Mycorrhizal inoculation markedly improved photosynthetic rate, and other gas exchange traits, chlorophyll content, and water use efficiency in *Ocimum basilicum* L. under saline conditions (Elhindi et al., 2017). AMF-inoculated *Allium sativum* plants showed improved growth traits including leaf area index, and fresh and dry biomass under saline conditions (Borde et al., 2010). Recently, Wang et al. (2018) have reported considerable enhancement in fresh and dry weights, and N concentration of shoot and root due to mycorrhizal inoculation under moderate saline conditions.

Furthermore, plants possessing AMF show enhanced synthesis of jasmonic acid, salicylic acid, and several important inorganic nutrients. For example, concentrations of total P, Ca^2+^, N, Mg^2+^, and K^+^ were higher in the AMF-treated *Cucumis sativus* plants compared with those in the uninoculated plants under salt stress conditions (Hashem et al., 2018). Mycorrhizal inoculation to *Capsicum annuum* exhibited enhanced chlorophyll contents, and Mg^2+^ and N uptake coupled with reduced Na^+^ transport under saline conditions (Cekic et al., 2012). In addition, Santander et al. (2019) have shown with lettuce that the mycorrhizal plants had higher biomass production, increased synthesis of proline, increased N uptake, and noticeable changes in ionic relations, particularly reduced accumulation of Na^+^, than those in non-mycorrhizal plants under stress conditions. AMF inoculation can effectively regulate the levels of key growth regulators. For example, Hameed et al. (2014) and Talaat and Shawky (2014) have observed AMF-mediated improvement in cytokinin concentration resulting in a marked photosynthate translocation under salinity stress. In addition, AMF-mediated growth promotion under salinity stress was shown to be due to alteration in the polyamine pool (Kapoor et al., 2013). Furthermore, Aroca et al. (2013) showed that enhanced strigolactone in AMF-treated plants notably mitigated various salinity effects in lettuce plants. AMF-colonized plants have the ability to decrease oxidative stress by suppressing lipid membrane peroxidation under salinity stress (Abdel Latef and Chaoxing, 2014; Talaat and Shawky, 2014). Furthermore, inoculation of AMF was also observed to enhance the accumulation of various organic acids resulting in up-regulation of the osmoregulation process in plants grown under saline stress. For example, Sheng et al. (2011) observed an enhanced synthesis/accumulation of certain organic acids in maize plants growing in saline soil, and AMF induced increased production of betaine, confirming the indirect role of AMF in plant osmoregulation under salinity stress.

### Heavy Metals

AMF are widely believed to support plant establishment in soils contaminated with heavy metals, because of their potential to strengthen defense system of the AMF mediated plants to promote growth and development. Heavy metals may accumulate in food crops, fruits, vegetables, and soils, causing various health hazards (Liu et al., 2013; Yousaf et al., 2016). AMF association with wheat positively increased nutrient uptake under aluminum stress (Aguilera et al., 2014). Plants grown on soils enriched with Cd and Zn exhibit considerable suppression in shoot and root growth, leaf chlorosis, and even death (Moghadam, 2016). There are many reports in the literature on uncovering the AMF-induced effects on the buildup of metals in plants (Souza et al., 2012; **Table 1**). Heavy metals can be immobilized in the fungal hyphae of internal and external origin (Ouziad et al., 2005) that have the ability to fix heavy metals in the cell wall and store them in the vacuole or may chelate with some other substances in the cytoplasm (Punamiya et al., 2010) and hence reduce metal toxicity in the plants. The strong effects of AMF on plant development and growth under severe stressful conditions are most often due to the ability of these fungi in increasing morphological and physiological processes that increase plant biomass and consequently uptake of important immovable nutrients like Cu, Zn, and P and thus reduced metal toxicity in the host plants (Kanwal et al., 2015; Miransari, 2017). It is also believed that enhanced growth or chelation in the rhizospheric soil can cause metal dilution in plant tissues (Kapoor et al., 2013; Audet, 2014). AMF reportedly bind Cd and Zn in the cell wall of mantle hyphae and cortical cells, thereby restricting their uptake and resulting in improved growth, yield, and nutrient status (Andrade and Silveira, 2008; Garg and Chandel, 2012).

Mycorrhizae can disturb the uptake of different metals into plants from the rhizosphere and their movement from the root zone to the aerial parts (Dong et al., 2008; Li et al., 2015). Mycelia of various AMF have a high cation-exchange capacity and absorption of metals (Takács and Vörös, 2003). Metal non-adapted AMF settle the polluted soils and reduce uptake and accumulation of heavy metals, as observed in perennial ryegrass (*Lolium perenne*) in artificially polluted soil with various elements like Cd, Ni, and Zn (Takács and Vörös, 2003). AMF are believed to regulate the uptake and accumulation of some key inorganic nutrients. For example, enhanced uptake of Si has been reported in mycorrhiza-inoculated plants like *Glycine max* (Yost and Fox, 1982) and *Zea mays* (Clark and Zeto, 2000). Hammer et al. (2011) also recorded considerable uptake of Si in spores and hyphae of *Rhizophagus irregularis* and its transfer to the host roots. It is pertinent that low Cd mobility and toxicity can also be addressed with AMF by increasing soil pH (Shen et al., 2006), restoring Cd in the extra-radical mycelium (Janouškova and Pavlíková, 2010), and binding Cd to glomalin, a glycoprotein. For example, in rice, AMF were very effective in lowering the levels of Cd in both the vacuoles and cell wall, which brought about Cd detoxification (Li et al., 2016a). Wang et al. (2012) observed that AMF-mediated improved Cd tolerance in alfalfa (*Medicago sativa* L.) had been possibly due to the modification of chemical forms of Cd in different plant tissues. Various processes that occur through the AMF are immobilization/restriction of metal compounds, precipitation of polyphosphate granules in the soil, adsorption to fungal cell wall chitin, and heavy metal chelation inside the fungus (**Figure 1**).

### Temperature (High and Low)

**Figure 2:**
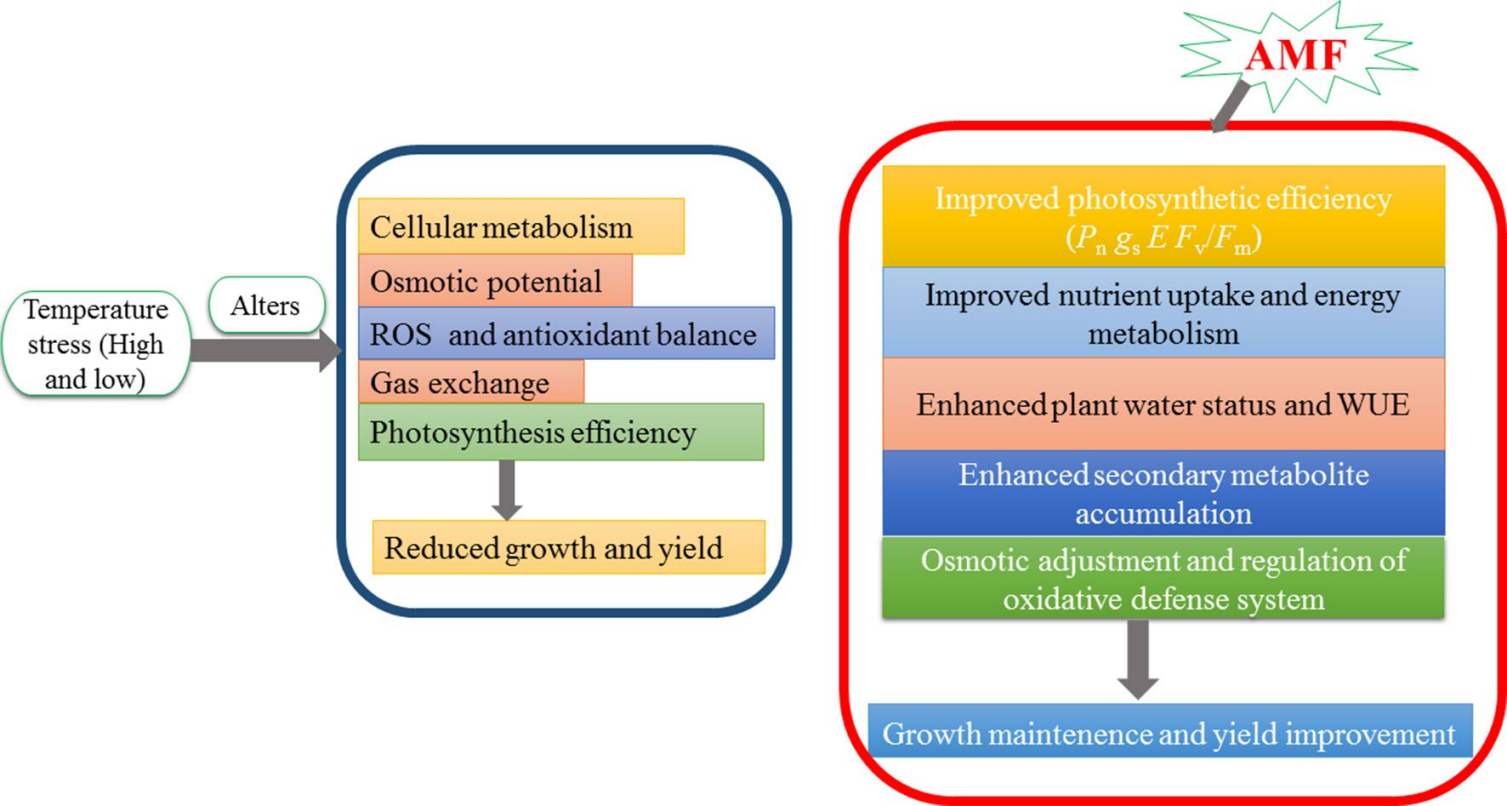
AMF inoculation alleviates temperature stress in plants.

As soil temperatures increase, plant community reactions may be dependent on AMF interactions for sustainable yield and production (Bunn et al., 2009). Heat stress significantly affects plant growth and development by imparting i) loss of plant vigor and inhibition of seed germination, ii) retarded growth rate, iii) decreased biomass production, iv) wilting and burning of leaves and reproductive organs, v) abscission and senescence of leaves, vi) damage as well as discoloration of fruit, vii) reduction in yield and cell death (Wahid et al., 2007; Hasanuzzaman et al., 2013), and viii) enhanced oxidative stress. Generally, AMF-inoculated plants show better growth under heat stress than do the non-AMF-inoculated ones (Gavito et al., 2005). Maya and Matsubara (2013) have reported the association of AMF *Glomus fasciculatum* with plant growth and development leading to positive changes in growth under the conditions of high temperature (**Figure 2**; **Table 1**).

AMF can increase plant tolerance to cold stress (Birhane et al., 2012; Chen et al., 2013; Liu et al., 2013). Moreover, a majority of reports state that various plants inoculated with AMF at low temperature grow better than non-AMF-inoculated plants (Zhu et al., 2010b; Abdel Latef and Chaoxing, 2011b; Chen et al., 2013; Liu et al., 2013). AMF support plants in combating cold stress and eventually improve plant development (Gamalero et al., 2009; Birhane et al., 2012). Moreover, AMF also can retain moisture in the host plant (Zhu et al., 2010a), increase plant secondary metabolites leading to strengthen plant immune system, and increase protein content for supporting the plants to combat cold stress conditions (Abdel Latef and Chaoxing, 2011b). For example, during cold stress, AMF-inoculated plants showed an enhanced water conservation capacity as well as its use efficiency (Zhu et al., 2010b). Symbiotic AMF relationship improves water and plant relationships and increases gas exchange potential and osmotic adjustment (Zhu et al., 2012). AMF improve the synthesis of chlorophyll leading to a significant improvement in the concentrations of various metabolites in plants subjected to cold stress conditions (Zhu et al., 2010a; Abdel Latef and Chaoxing, 2011b). The role of AMF during cold stress has also been reported to alter protein content in tomato and other vegetables (Abdel Latef and Chaoxing, 2011b).

## AMF and Combined Abiotic Stresses

It is widely accepted that AMF could alleviate various stresses or combination of stresses that include, drought, salinity, temperature, nutrients, and heavy metals. For example, exposure of plants to a combination of drought and salinity causes an enhanced production of reactive oxygen species, which can be highly injurious to plants (Bauddh and Singh, 2012). Detoxification of reactive oxygen species (ROS) is done by the enzymes that include commonly superoxide dismutase (SOD), catalase (CAT), peroxidase (POD), and glutathione reductase (GR) (Ahanger and Agarwal, 2017). In addition, combined application of drought and salinity to tomato plants inoculated with *Scolecobasidium constrictum* showed improved biomass production, leaf water relations, stomatal conductance, and *F*v/*F*m relative to those in non-inoculated plants (Duc et al., 2018). Thus, AMF are critical for improving plant growth and yield under stress (Abdel Latef, 2011; Abdel Latef and Chaoxing, 2011a; Abdel Latef and Chaoxing, 2011b; Abdel Latef and Chaoxing, 2014). Very rare research reports are available in the literature demonstrating the role of AMF in mitigation of combined effects of two or more stresses. AMF symbiosis protects plants against a variety of abiotic stresses using various processes such as improved photosynthetic rate, uptake and accumulation of mineral nutrients, accumulation of osmoprotectants, up-regulation of antioxidant enzyme activity, and change in the rhizosphere ecosystem (Bárzana et al., 2015; Calvo-Polanco et al., 2016; Yin et al., 2016). Several studies have shown improved nutritional status of AMF plants under osmotic stress conditions (Augé et al., 2014; Lehmann et al., 2014; Lehmann and Rillig, 2015) resulting from deficit irrigation or salinity. Similarities among the tolerance mechanisms may occur in response to AMF-mediated combined stress adaptations. It is proposed that AMF-mediated alterations in phytohormone profile, mineral uptake and assimilation, accumulation of compatible osmolytes and secondary metabolites, and up-regulation of antioxidant system can be the common mechanisms induced during different stresses. However, specific mechanisms like compartmentation and sequestration of toxic ions, production of phytochelatins, and protein expression can be specific and exhibit a significant change with stress type and the AMF species involved. Changes in root characteristics like hydraulic conductivities can improve the osmotic stress tolerance to considerable levels (Evelin et al., 2009). Zhang et al. (2018b) have shown that the AMF protected castor bean against saline stress by altering gas exchange traits and the levels of some key metabolites. The said characteristics of AMF may elevate nutraceutical quality of crops and could be of considerable agronomic importance for production and management of different potential crops. However, extensive studies are required to unravel the role of AMF in counteracting the effects of combined stresses.

## Conclusion and Future Prospects

A few research reports have already documented the beneficial role of AMF in improving plant growth under stressful environments. Therefore, in this review, the existing information related to the role of AMF has been combined in a coherent way for understanding of AMF symbiotic relationship with a variety of plants under stress environments. Previously, the AMF have been mainly discussed as beneficial entities for nutrient uptake from soil; however, recently, it has been clearly depicted that plants inoculated with AMF can effectively combat various environmental cues, like salinity, drought, nutrient stress, alkali stress, cold stress, and extreme temperatures, and thus help increase per hectare yield of a large number of crops and vegetables. Encouragement of AMF usage is of immense importance for modern global agricultural systems for their consistent sustainability. Undoubtedly, exploitation of AMF for agricultural improvement can significantly reduce the use of synthetic fertilizers and other chemicals, thereby promoting the bio-healthy agriculture. AMF-mediated growth and productivity enhancement in crop plants can be beneficial to overcome the consumption requirement of increasing population across the globe. In addition, environment-friendly technologies shall be highly encouraged due to their widespread use. The primary focus of future research should be on the identification of genes and gene products controlling the AMF mediated growth and development regulation under stressful cues. Identification of both host as well as AMF specific protein factors regulating symbiotic association and the major cellular and metabolic pathways under different environmental stresses can be hot areas for future research in this field. Understanding the AMF induced modulations in the tolerance mechanisms and the crosstalk triggered to regulate plant performance can help improve crop productivity. Taken together, AMF must be explored at all levels to further investigate their role in nature as a bio-fertilizer for sustainable agricultural production.

## Author Contributions

NB, CQ, MAA, SR, MIK, NA, and LZ contributed equally in preparation of this manuscript. MA helped considerably in writing of this manuscript and made final corrections.

## Funding

This work was supported by the National Key Research and Development Program of China (2017YFE0114000), Sci-tec Project of China Tobacco Shaanxi Industrial Co. Ltd. (SXYC-2016-KJ-02) and Sci-tec Project of Shaanxi China Tobacco Industrial Co., Ltd. (JS-FW-2016-001).

## Conflict of Interest Statement

All authors declare that there is no potential conflict of interest with any commercial or financial institution other than acknowledged in “Funding” section of this manuscript.
